# A Method for Real-Time Measurement of the Vertical Vortex at Flood Discharge Outlets Using Ultrasonic Sensors

**DOI:** 10.3390/s24175583

**Published:** 2024-08-28

**Authors:** Dingfan Fan, Min Yu, Zhixiang Yao, Yang Du, Hang Liu

**Affiliations:** 1 School of Naval Architecture Ocean and Energy Power Engineering, Wuhan University of Technology, Wuhan 430063, China; findingfun@whut.edu.cn (D.F.); 284477@whut.edu.cn (Y.D.); hangliu@whut.edu.cn (H.L.); 2 College of Electronic Engineering, Naval University of Engineering, Wuhan 430030, China; whyaozx@163.com

**Keywords:** acoustic measurement, ultrasonic sensor, vortex acoustic coupling, vertical vortex, Particle Image Velocimetry, Computational Fluid Dynamics method

## Abstract

In this study, ultrasonic sensors were used to measure the vertical vortex at flood discharge outlets in real time, and numerical simulations and model experiments were conducted. When a sound signal passes through a vortex, its propagation characteristics will change, which helps to determine the existence of the vortex. Moreover, its characteristic parameters can be obtained through inversion. In this paper, first, the theories of acoustic measurement methods were introduced and their feasibility was verified through a comparison between Particle Image Velocimetry (PIV) measurement and numerical simulation results. Then, the Computational Fluid Dynamics (CFD) method was used to simulate the vertical vortex at the flood discharge outlets of hydraulic structures and the simulation data were restored to the actual size at scale. Finally, acoustic numerical simulations of actual vortex data were conducted, and ultrasonic sensors were used to measure the velocity of a simplified vertical vortex model under laboratory conditions. The research results indicate that the acoustic measurement method proposed in this article is effective in the measurement of the characteristic parameters of vertical vortex with a core radius of 0.03~0.05 m and a maximum tangential velocity of 0.5 m/s, the measurement error of the maximum tangential velocity is within 10%.

## 1. Introduction

Hydraulic structures play an important role in water resource management, flood control and disaster reduction, and energy production. Flood discharge outlets constitute a key component of hydraulic structures [1]. The flood discharge outlet serves as both a regulator of the water level of a reservoir and a discharge port for excess water, ensuring safe drainage during floods and flood seasons. During the flood discharge process, the flow pattern of water is complex and variable, and a series of harmful hydraulic phenomena, such as a vertical vortex, will occur at the flood discharge outlet [2].

The vertical vortex is a strong rotating water flow formed under specific conditions, and it may lead to serious safety issues and economic losses in engineering [3,4,5]. A proper means to monitor the real-time velocity of the vortex at the flood discharge outlet will enable the management of the velocity, thereby improving our understanding of the vertical vortex and promoting the development of effective monitoring and control methods.

Significant breakthroughs made in non-invasive velocity measurement technology since the 1980s, especially the emergence of Laser Doppler Anemometry (LDA) and Particle Image Velocimetry (PIV), have been a great aid to the study of the vertical vortex. Ru et al. (1992) measured the distribution of the vertical vortex flow field using a two-dimensional Doppler velocimeter [6]. Yingkui (2011) conducted experimental research on the hydraulic model of Xiluodu Power Station using a Velocity Dynamic Measurement System (VDMS) [7]. Rajendran and Constantescu et al. (2001–2003) conducted experiments on the vertical vortex in a water pump suction pool with the help of PIV technology [8,9,10]. They measured the velocity distribution and analyzed the internal dynamics of the vertical vortex. They also investigated the structure of the vertical vortex on a two-dimensional plane. Miao (2019) used the PIV method to study the vortex in different flood discharge outlet models. The results showed that the number and intensity of the vortexes decreased with the increase of the water depth on the measured cross-sections of the free surface [11], sidewall, and bottom plate. Yunpeng (2022) proposed a flow field observation algorithm based on the Horn–Schunck (HS) method, and used it in combination with a physical model for the observation of the surface flow morphology during the formation and evolution of the vertical vortex in a certain dam section [12]. It was found that the optical flow method can collect more information and better track large vertical vortexes, compared with traditional PIV technology.

However, the above-mentioned observation and research methods have limitations in measuring the vertical vortex at the discharge outlet of actual hydraulic structures. For example, the LDA and PIV methods have high requirements for light intensity [13,14]. Due to the substantial attenuation of light propagation in water, the propagation distance is short, making it difficult for large-scale measurements. In addition, particle addition to the tested water area in the PIV method also causes pollution to the environment.

Compared to light, sound has far better underwater propagation performance, and it is currently the only information carrier capable of long-distance propagation. Acoustic detection has the advantages of being global and non-invasive [15]. Acoustic methods address the limitations of the existing measurement methods of the vortex. The acoustic measurement experiment of the underwater vortex flow field mainly revolves around ultrasonic detection technology of the vortex. Lillberg (2000) conducted model experiments on sound propagation in the vortex with the aim of developing underwater ultrasonic testing technology of the vortex field [16]. Christopher et al. (2004) studied the sources of cavitation sounds using the coupling effect of the vortex on acoustic signals in the Garield Thomas Waterway in Pennsylvania, USA [17,18]. The experiment proved that compared with traditional underwater measurement techniques, the acoustic detection method had better performance. Rosny and Tourin (2005) addressed the problem of difficulty in measuring small phase jumps in acoustic signals caused by a low Mach number vortex flow [19]. They used Time Reverse Mirror (TRM) technology to enlarge the phase jumps and reduce interference. They finally acquired accurate phase data and achieved good results. Zhao, Heming, et al. (2018) conducted numerical simulations to investigate the disturbance effect caused by ultrasonic flow meter probes and calculated the correction coefficient for laboratory calibration [20]. They quantitatively analyzed the distribution of channel velocity, the probe sound pressure signals, and the relationships between the two.

In addition to experimental methods, Computational Fluid Dynamics (CFD) methods are also widely used in the study of the vertical vortex. With the advantages of low cost, fast analysis, and complete information, numerical simulation can directly simulate the vertical vortex generated by the prototypes of hydraulic structures. Constantinescu (2000) used two turbulence models to numerically simulate the inlet of a water pump [21]. The two turbulence models achieved consistent results in the simulation of the shape and size of the vortex. However, there were some differences in the simulated position and intensity of the vortex between the two models. Biao and Li (2003–2004) handled the free liquid surface of a vertical vortex based on the solid lid assumption and numerically modeled and simulated the inlet of a water pump [22,23,24]. Sakai’s team (2008) developed a vertical vortex prediction method based on the Burgers vortex model [25]. This method calculates the circulation and downward velocity gradient through single-phase simulations and uses these two parameters to estimate the length of the core of the vertical vortex. Frank (2014) used the fused deposition modeling (FDM) method to calculate the effects of surface tension, viscosity, and turbulence on the free surface depression of a vertical vortex [26]. Huang (2021) employed the Volume of Fluid (VOF) method to numerically simulate the hydraulic characteristics of the vortex at the flood discharge outlet of a large hydropower station [27]. This study clarified the causes and trends of the vortex in front of the dam, and the impact of the vortex on the flood discharge structure of the dam. Moreover, it proposed a scheduling plan to weaken the harmful vortex in the surface hole, gate slot, and gate head.

The VOF method has been extensively applied in numerical simulations to solve the problem of complex two-phase flows on the free surface of a vertical vortex, and achieved good results. In recent years, many application examples have been reported in the numerical simulation of the vertical vortex [28,29,30]. In the present article, the VOF method was also used to simulate relevant models.

To better understand and measure the vertical vortex, an acoustic measurement method was proposed for the vertical vortex in the present paper. This article is organized as follows. Section 2 introduces the theories of applying the acoustic measurement method to the vertical vortex, constructs a vertical vortex model based on the flow velocity measured by PIV, and makes numerical simulations to verify the feasibility of the method. In Section 3, CFD simulation is used to obtain the flow velocity at the flood discharge outlet of a real hydraulic building. In Section 4, acoustic numerical simulations of the velocity are carried out. By comparing the CFD velocity with the inversion velocity, it is verified that the acoustic measurement method can be applied to real-time measurement of the velocity parameters of the vertical vortex at the flood discharge outlet. In addition, a vertical vortex model is also established in a laboratory water tank, and ultrasonic sensors are used to measure the vertical vortex velocity at a constant flow rate and different water levels. The experiments prove that the acoustic measurement method proposed is useful in the measurement of the velocity of the vertical vortex. Section 5 draws conclusions and viewpoints, and highlights the wide prospects of the acoustic measurement method proposed in this article.

## 2. Principles of the Acoustic Measurement Method

### 2.1. Equations for the Vertical Vortex

The basic equations of the vertical vortex include the Navier–Stokes equation describing fluid motion in fluid dynamics and its simplified form under specific conditions. The simplified form is often used to find a solution that can approximate the motion characteristics of the vertical vortex.

Both theoretical analysis and experimental observations have shown that axisymmetric vertical vortexes are formed at the outlet whether it is at the bottom or one side. Experimental research on the vertical vortex mainly focuses on the variation patterns of its velocity, pressure, and vorticity fields. Bernoulli’s theorem explains the relationship between pressure and velocity fields. Vorticity is defined as the curl of the velocity field. Therefore, in vertical vortex experiments, the most crucial step is to acquire the velocity distribution.

The Rankine vortex model is one of the classic vortex theoretical models [31]. It defines the radius at the maximum tangential velocity $V_{\theta -\max}$ of the vortex as the vortex core radius R. The velocities inside and outside the vortex core radius are both related to the maximum tangential velocity.

(1) $$\left\{ \begin{array}{l} V_{\theta }=V_{\theta -\max}\cdot \frac{r}{R},r\leq R\\ V_{\theta }=V_{\theta -\max}\cdot \frac{R}{r},r\geq R \end{array}\right.$$

**Figure 1:**
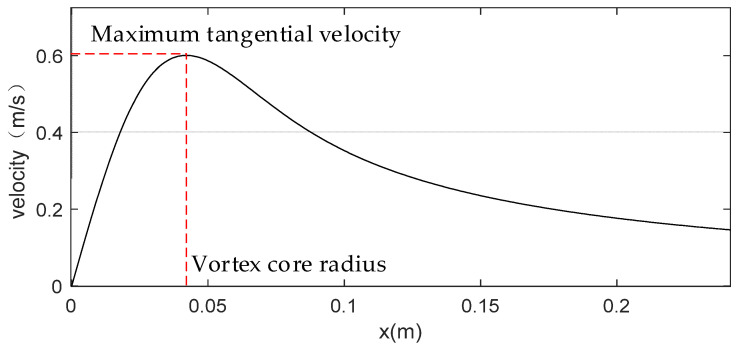
Velocity distribution of the Rankine vortex model. The maximum tangential velocity and vortex core radius of the vortex are evident in the figure.

Therefore, based on the measurements of the maximum tangential velocity and the vortex core radius by an appropriate method, the theoretical velocity distribution of the vortex can be constructed (Figure 1).

### 2.2. The Acoustic Coupling Effect of the Vortex

**Figure 2:**
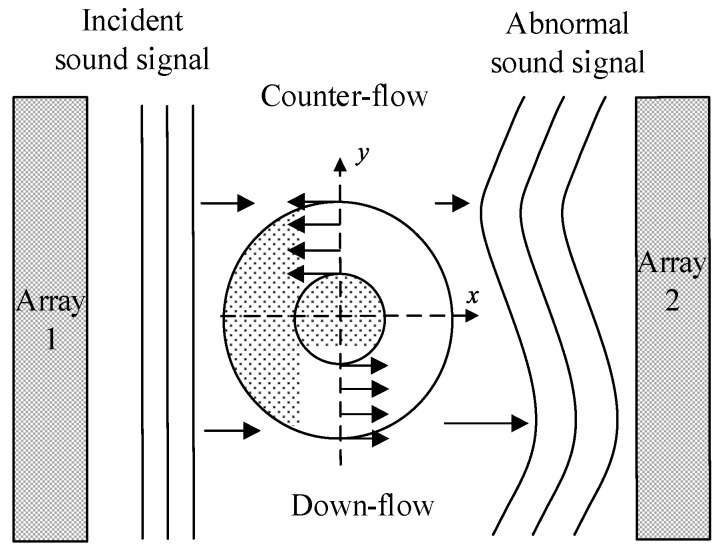
Schematic diagram of sound propagation characteristics in the vortex. Array 1 is the transmitter array, and array 2 is the receiver array.

As sound signals pass through the vortex, the propagation characteristics are affected by the internal flow structure of the vortex, and the changes in sound signal are closely related to the morphological characteristics of the vortex [32,33,34]. Specifically, the amplitude and phase of sound signals change after they pass through the vortex. As shown in Figure 2, the sound signal accelerates in the down-flow region, leading to a phase lead in signal receiving and a shorter propagation time. The deceleration of sound signals in the counter-flow zone results in a phase lag in signal receiving and a longer propagation time. Consequently, different time delays (i.e., changes in propagation time) are produced.

Assuming that in the absence and presence of the vortex, the time required for the sound signal to propagate from array 1 to array 2 is $t_{1}$ and $t_{2}$, respectively, the time delay of the sound signal is calculated by $\Delta t=t_{2}-t_{1}$. The propagation time of the sound signal in the absence and presence of the vortex is:

(2) $$t_{1}=\int_{1}^{2}\frac{dx}{c}$$

(3) $$t_{2}=\int_{1}^{2}\frac{dx}{\left(c+u\cdot n\right)}$$

In Equations (2) and (3), c represents the sound velocity in water, which is set at 1500 m/s. Arrays 1 and 2 denote the transmitter and receiver of the sound signal, respectively. ***u*** is the vortex velocity, and **n** is the position corresponding to this velocity. The time delay difference of sound signals propagating in the vortex is expressed as:

(4) $$\Delta t=t_{2}-t_{1}=\left(\int_{1}^{2}\frac{dx}{\left(c+u\cdot n\right)}-\int_{1}^{2}\frac{dx}{c}\right)$$

Expanding Equation (4) into a series and neglecting higher-order terms yields:

(5) $$\Delta t=\frac{1}{c^{2}}\int_{1}^{2}u\cdot ndx$$

Equation (5) reveals the correlation between the time delay of the sound signal passing through the vortex and the vortex velocity. When the positions of the sound signal transmitter and receiver are fixed, and the time delay difference Δt caused by the sound coupling effect of the vortex is known, the velocity distribution of the vortex can be calculated.

Since velocity is a vector related to space, it is necessary to use the velocity field reconstruction algorithm to invert the velocity distribution of the vortex for the collection of the measured vortex flow field information, such as the magnitude and distribution of velocity [35]. It is the basic idea of the acoustic measurement method proposed for the vertical vortex in this article.

### 2.3. Validation by the PIV Method

PIV technology has high precision and resolution and can detect the transient distribution of planar velocity [36]. In the present study, the velocity of a vertical vortex model was measured using the PIV method, and the acoustic measurement method was used to simulate the data. The velocity simulated by the acoustic measurement method was compared with that measured by the PIV method to verify the accuracy of the proposed method.

#### 2.3.1. Measurement Results of the PIV Method

**Figure 3:**
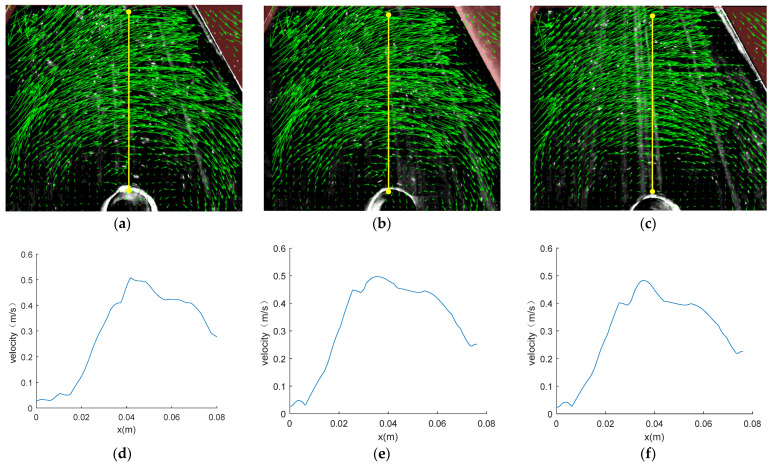
Flow velocity measured by PIV. (**a**), (**b**), and (**c**) show the velocity vectors measured by PIV at z = 0.03 m, z = 0.05 m, and z = 0.07 m, respectively. (**d**), (**e**), and (**f**) show the velocity distribution curves at z = 0.03 m, z = 0.05 m, and z = 0.07 m, respectively. (**a**) Velocity vector at z = 0.03 m; (**b**) Velocity vector at z = 0.05 m; (**c**) Velocity vector at z = 0.07 m; (**d**) Velocity distribution curve at the yellow line on the plane of z = 0.03 m; (**e**) Velocity distribution curve at the yellow line on the plane of z = 0.05 m; (**f**) Velocity distribution curve at the yellow line on the plane of z = 0.07 m.

The experiment was conducted in a specially designed circulating water channel, and details are shown in Section 4.2. Measurements were taken with the water surface as the reference plane. The velocity distribution maps at different depths (z) are plotted in Figure 3. Figure 3a–c show velocity vectors measured by PIV. The size and direction of each vector represent the magnitude and direction of the velocity at that point, respectively. The curves in Figure 3d–f represent the velocity distribution on the plane derived at the corresponding line segment.

The PIV method can measure the global velocity on the plane. It can be seen from velocity vector figures that the flow velocity decreases as it approaches the center of the vortex. Similarly, on the velocity distribution curve, the flow velocity of the vortex is extremely low at x = 0~0.01 m, increases with increasing x, reaches its maximum near x = 0.04 m, and then decreases as x continues to increase.

The maximum tangential velocity and vortex core radius on different planes measured by PIV were derived and the results are shown in Table 1.

#### 2.3.2. Comparison and Analysis for the PIV Method

The acoustic measurement method was used for numerical simulation of the velocity distribution of the vortex measured by the PIV method. Considering that the vertical vortex is stable and axisymmetric, the tangential velocity was used to describe its velocity distribution and the V4 spline interpolation technique was employed to supplement the missing data.

**Figure 4:**
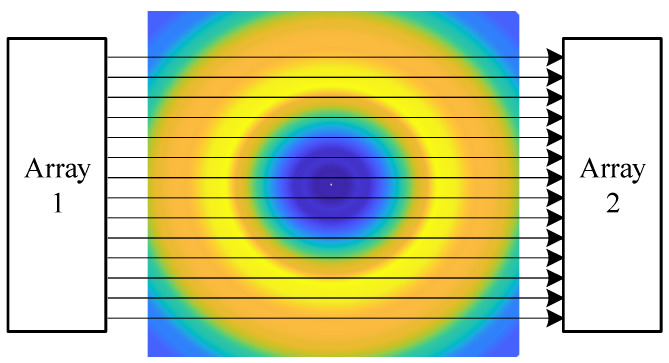
Schematic diagram of numerical simulation using the acoustic measurement method. Array 1 is the transmitter array and array 2 is the receiver array, with a vortex in the middle and arrows indicating the trajectory of the acoustic signal.

During the interpolation of the vortex velocity distribution, 100 pairs of ultrasonic transmitter/receiver arrays were installed on both sides of the vortex in the MATLAB simulation program to numerically simulate the time delay of each path (Figure 4). The vortexes on all planes were simulated in order.

**Figure 5:**
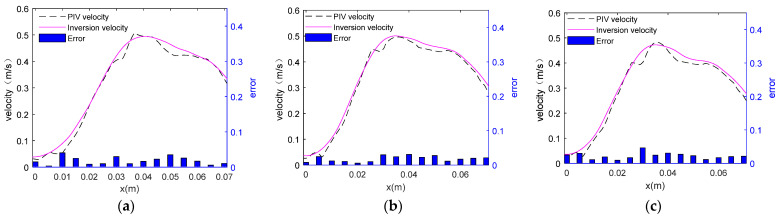
Biaxial column diagram of the velocity distribution and absolute error on different measurement planes. (**a**) Velocity data on the plane of z = 0.03 m; (**b**) Velocity data on the plane of z = 0.07 m; (**c**) Velocity data on the plane of z = 0.07 m.

The velocity measured by PIV and the inversion velocity were displayed in the same coordinate system, and the absolute error between them was calculated. Figure 5 shows them on different planes.

As shown in Figure 5a–c, the change trend of the velocity simulated by the acoustic measurement method is basically consistent with that measured by the PIV method. It is obvious that the velocity gradually increases with the increase of the x coordinate value from 0, and then slowly decreases after it reaches the maximum tangential velocity (where x = vortex core radius). Thus, there are a forced vortex zone and a free vortex zone in the curve. This feature is consistent with that of the ideal Rankine vortex model.

According to the Rankine vortex model, the key parameters in vortex investigation are its maximum tangential velocity and the vortex core radius. Therefore, the velocity data obtained by the two methods were compared and the PIV data were used as the standard value in the calculation of the error of the data measured by the acoustic measurement method. The data are shown in Table 2 and Table 3.

In Table 2, the absolute and relative errors between the data simulated by the acoustic measurement method and measured by PIV are less than ±0.01 and 10%, respectively. This indicates that the acoustic measurement method can effectively measure the vertical vortex velocity, and the measurement error for the maximum tangential velocity is small. The results verified the feasibility of the acoustic measurement method.

## 3. CFD Simulation of the Vertical Vortex

The Yangtze River is the golden waterway with the highest freight volume among the world’s inland waterways, and it plays an important strategic role in the development of the western region of China. The Three Gorges Water Conservancy Hub Project is located in Hubei Province, China, known as one of the largest water conservancy and power generation projects in the world at present.

CFD is a powerful tool that can simulate and display the fluid motion of a vertical vortex in three-dimensional space in detail with the help of FLUENT (in Workbench 2023 R1). Due to practical factors such as flood control scheduling and dam protection, it is impossible to carry out on-site experimental verifications at a real flood discharge outlet. Therefore, CFD simulation of the flood discharge outlet of the Three Gorges Dam was carried out and the original size of the vortex was restored to scale. Finally, the vertical vortex forming in a real hydraulic structure was simulated.

### 3.1. Prototype of the Hydraulic Structure

The entire Three Gorges Dam spans the Yangtze River, with the total axis length being 2309 m, flood discharge segment length being 483 m, dam crest elevation being 185 m, maximum dam height being 181 m, and water storage elevation being 175 m. The flood discharge section is located in the middle of the riverbed, where there are 23 deep holes and 22 surface holes [37].

The artificial lake formed upstream of the Three Gorges Dam is called the Three Gorges Reservoir, which has a total storage capacity of $393\times 10^{9}\;m^{3}$. When the flood season approaches, through the flood control operation of the reservoir, the peak flow can be reduced by $2.7\times 10^{4}\;m^{3}/s$ to $3.3\times 10^{4}\;m^{3}/s$. This helps effectively control floods in the upper reaches of the Yangtze River.

**Figure 6:**
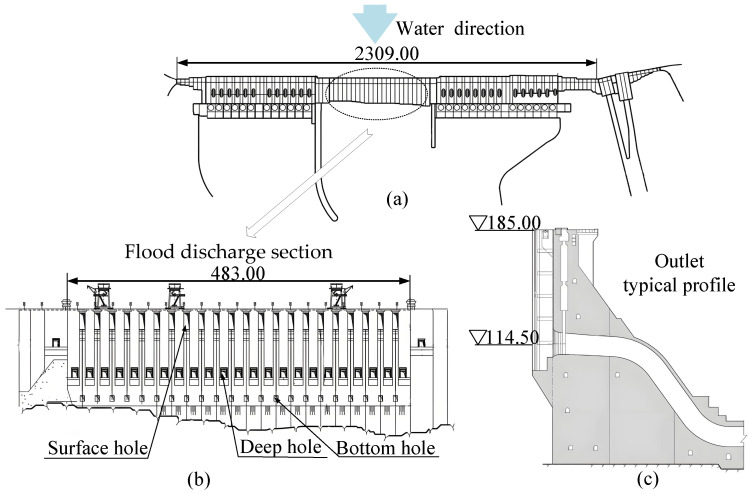
Schematic diagram of the overall structure of the Three Gorges Dam: (**a**) shows the layout plan of the dam, (**b**) shows the flood discharge outlet, and (**c**) shows the typical cross-section of the flood discharge outlet.

As shown in Figure 6a, the flood discharge section of the Three Gorges Dam is flat and unobstructed, which meets the conditions for acoustic measurement.

### 3.2. CFD Simulation

#### 3.2.1. Model Setting and Mesh Division

The Three Gorges Dam was simulated and modeled at a 1:100 scale, and the model has a length of 23.00 m and a height of 1.85 m. The simulation area is from 30.00 m in front of the dam to 10.00 m behind the dam, and the research focus is the vertical vortex at the flood discharge outlet. The scales of the model are detailed in Table 4.

After the calculation domain was determined, the upstream water level was set at 1.75 m according to the practical situation of engineering. The upstream reservoir was taken as the velocity inlet and the flood discharge outlet as the pressure outlet. The pressure was set at 1 atm pressure. At the water–air interface, the pressure is set to be equal to atmospheric pressure and the boundary condition at solid walls is specified as a no-slip condition. The standard wall function was used to solve the flow in the near-wall region.

**Figure 7:**
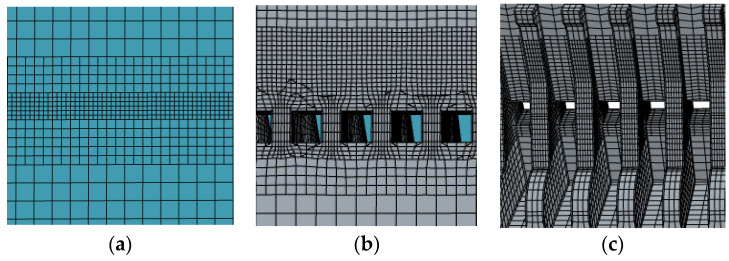
Schematic diagram of mesh division in different regions. (**a**) Flow field calculation domain; (**b**) Flood discharge inlet; (**c**) Flood discharge outlet.

In the process of mesh division, the use of a hexahedral mesh can reduce a large number of meshes and improve computational efficiency. The structure of the flood discharge section is complex, with partition piers arranged between stacked beam doors and horizontal beams arranged at the inlet. Therefore, the region between the inlet and outlet was subject to mesh refinement, while the others area was divided into less dense meshes [38]. Mesh division is introduced in Figure 7.

Ansys Fluent software was used for CFD simulation, and a static flow field was simulated first. To simulate the free surface vortex at the water–air interface of the vertical vortex, the VOF method was introduced. The VOF method is an interface tracking technique based on the Euler method, mainly used to simulate free surface flows in multiphase flows. The key of the VOF model is to track the distribution of each phase by defining a volume fraction function, which represents the volume proportion occupied by a specific phase in a computing unit. By directly tracking the changes in fluid volume in each unit, the process of interface tracking is simplified. Meanwhile, the use of high-order difference schemes and reconstruction algorithms can help achieve high numerical accuracy.

#### 3.2.2. Simulation Results

**Figure 8:**
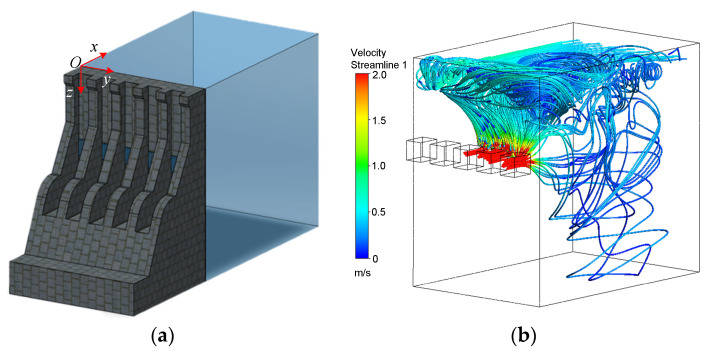
Flood discharge outlet model. (**a**) Flood discharge outlet model where a vertical vortex appears; (**b**) Velocity trajectory map of the vertical vortex at that location.

The simulation results showed that at 56.44 s, a vertical vortex appeared at the 3^rd^ to 5^th^ flood discharge outlets, with a flow velocity ranging between 0 m/s and 0.7 m/s and a vortex scale of 0.03 m to 0.15 m. A velocity trajectory map was drawn at this location (Figure 8).

As shown in Figure 8b, the incoming flow is affected by the incoming flow and boundary conditions at the 3^rd^ to 5^th^ outlets, resulting in the formation of a vertical vortex.

**Figure 9:**
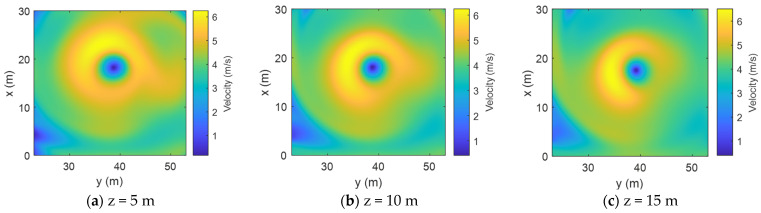
Velocity cloud map of the vertical vortex at the flood discharge outlet on planes of different depths (the velocities in the figure are in all directions).

The actual size and flow velocity of the vertical vortex were restored at scale, as shown in Table 3, and the data were supplemented by the V4 spline interpolation method. Velocity cloud maps were drawn at different depths (z) from the horizontal plane, which was taken as the *x-O-y* plane, with an interval of 5 m (Figure 9). The velocity parameters of the vortex are listed in Table 5.

According to Figure 9, on the same plane, the tangential velocities of the vertical vortex are distributed in a circular pattern around the vortex core, and the vertical vortex can be approximately equivalent to an axisymmetric uniform vortex. As the depth increases, the velocity distribution of the fluid changes, and the vortex core radius gradually decreases.

Furthermore, the vertical vortex parameters restored to scale were derived (Table 5). According to Table 4, the maximum tangential velocity ranges between 4 m/s and 6 m/s, and the vortex core radius ranges between 5 m and 10 m. The data provide vortex field parameters for the subsequent acoustic simulations.

## 4. Acoustic Simulation and Experiment

### 4.1. Numerical Simulation of the Acoustic Measurement Method

According to the position of the vertical vortex at the flood discharge outlet and the shape of the dam in the actual vortex data, signal receiver and transmitter arrays were placed at suitable positions. Meanwhile, an appropriate frequency and signal shape were selected. By measuring the time delay difference of the sound signal passing through the vertical vortex, a real-time monitoring method for the vortex at the flood discharge outlet was developed.

#### 4.1.1. Parameter Settings

Multiple factors were taken into account in the parameter settings of the sensor arrays.

1. The sensor array should not obstruct the drainage at the flood discharge outlet. To achieve this end, the sensors should be placed far away from areas with significant changes in flow velocity. Finally, the sensors were arranged on both sides of the upstream bank far from the flood discharge section, just like shore-based sonar.
2. The monitoring path of the sound signal should cover the area where the vertical vortex appears at the flood discharge outlet, which makes requirements for placement range of the sensor array. In the present study, a total of 300 sets of ultrasonic transmitter and receiver arrays were arranged at an interval of 0.1 m in the direction of the incoming flow from the dam, with the array length totaling 30 m. The depths were set at z = 5 m, z = 10 m, and z = 15 m.
3. During flood discharge, the dam may generate background noise due to vibration, water flow impact, etc. The natural vibration frequency of the dam is usually within 10 Hz [39], and the water flow noise frequency is between 20 Hz and 800 Hz. The underwater noise spectrum level at deep holes is between 10 and 30 dB [40,41]. To avoid diffraction, the wavelength of the sound signal should be significantly smaller than the vortex core radius. A pulse signal with a frequency of 50 kHz was finally used as the sound signal, including 20 cycles, with a trigger interval of 1 s.

**Figure 10:**
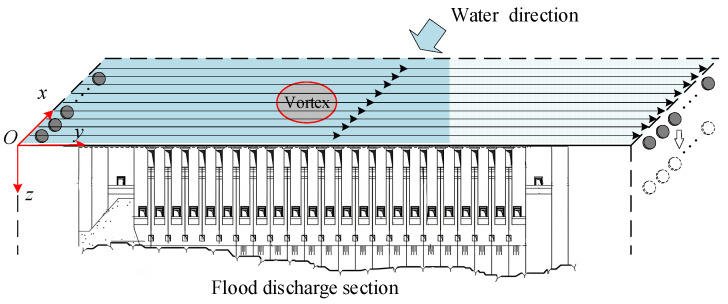
Schematic diagram of ultrasonic transmitter and receiver arrays placed at the flood discharge outlet.

**Figure 11:**
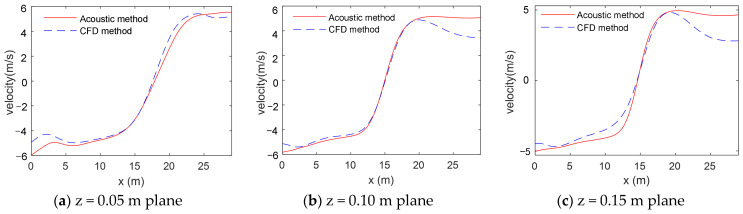
Velocity distribution curves of the vortex on different planes. The red solid line represents the inversion velocity, and the blue dashed line represents the CFD velocity.

The positions of the transmitter and receiver arrays for the sound signals in the acoustic simulation are shown in Figure 10. Sound signal transmission/reception ends were placed on both sides of the fluid to simulate the time delay difference Δt on each path. The vortexes on all the CFD simulation planes were simulated in sequence to obtain the time delays at different *x* coordinates. Then, the phase reconstruction algorithm was used to calculate the corresponding flow velocity **u** from Δt, and the velocity distributions on different planes were acquired (Figure 11).

With the increase of the z value, the fluctuations of the flow velocity curve are reduced in Figure 11, indicating that the tangential velocity slows inside the vortex. Moreover, the change trend of the velocity measured by the acoustic measurement method is consistent with that of the velocity simulated by CFD. The curve measured by the acoustic method shows the velocity change and also characterizes the velocity parameters of the vertical vortex, such as the maximum tangential velocity and vortex core radius. Specific data are summarized in Table 6.

#### 4.1.2. Comparison and Analysis

**Figure 12:**
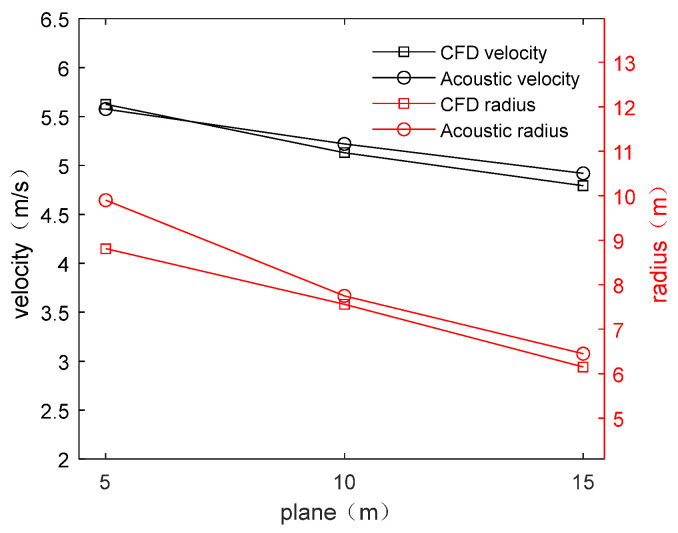
Line chart of vortex data. The black line in the figure represents the maximum tangential velocity obtained by the two methods, with their coordinate axes on the left. The red line represents the vortex core radius obtained by the two methods, with their coordinate axes on the right. Different methods are distinguished by □ and ○.

The most important parameters of the vertical vortex are the maximum tangential flow velocity and vortex core radius. The CFD simulation values and acoustic inversion values of the two parameters listed in Table 5 and Table 6 are plotted on the same line chart in Figure 12 for the analysis of their relationship.

It can be seen from Figure 12 that both the CFD-simulated and inversion parameters (i.e., maximum tangential velocity and vortex core radius) of the vertical vortex at the flood discharge outlet decrease with the increase of the depth of the measurement plane. Moreover, the data obtained by the two methods are relatively close. The errors between the CFD simulation data and acoustic inversion data were calculated, and the reasons for the errors were analyzed. It is proven that the acoustic measurement method proposed for the vertical vortex at the flood discharge outlet is feasible and has high accuracy.

According to Table 7, the absolute error in maximum tangential velocity of the vortex measured by the acoustic measurement method and the CFD simulation method is relatively small, with small variations that fall within ±0.1. The relative error of the maximum tangential velocity can be controlled within 10%, indicating that the acoustic measurement method has high accuracy in the measurement of the velocity parameters of the vertical vortex. The relative error of the vortex core radius is relatively large. The reason is that the vortex core radius decreases with the increase of the *z* value, resulting in an increase in relative error when the absolute error remains unchanged.

It is believed that the main reason for the error between the inversion velocity and the CFD-simulated velocity is that the phase reconstruction algorithm assumes that the vortex is uniform and axisymmetric with uniform velocity, while the velocity distribution simulated by CFD is not completely uniform. When the CFD simulation vortex is more similar to the ideal vortex, the error will be smaller.

The comparison between the inversion velocity and the CFD simulation velocity demonstrates that the acoustic measurement method can accurately reproduce the vortex in numerical simulation, and it has potential for practical applications.

### 4.2. Experimental Methods

Although it is impossible to conduct field measurements to study the vertical vortex at the flood discharge outlet, a simplified model of the vertical vortex was established in the laboratory water tank, and acoustic instruments were used to measure the flow velocity of the vortex. Moreover, the acoustic measurement results were compared with the PIV measurement results given in Section 2.3 to verify the feasibility and accuracy of the acoustic measurement method.

#### 4.2.1. Experimental Setup

**Figure 13:**
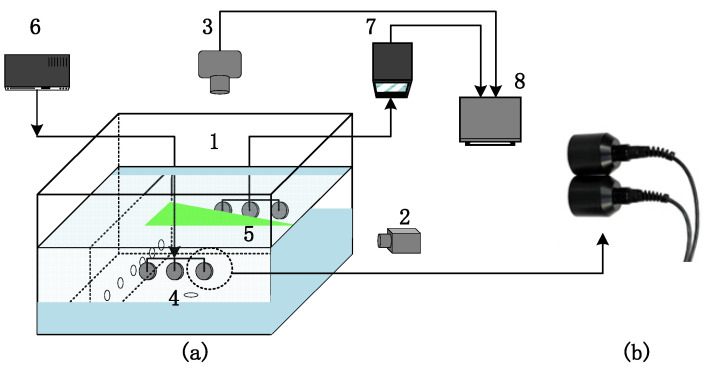
(**a**) is the schematic diagram of the experimental system. 1. Water tank; 2. Laser; 3. CCD camera; 4. Ultrasonic transmitter array; 5. Ultrasonic receiver array; 6. Signal generator; 7. Data acquisition instrument; 8. Data processing device. (**b**) shows the actual ultrasonic sensor used in this experiment.

This experiment was conducted in a customized circulating water channel, and the experimental system is shown in Figure 13. Instruments such as signal generator, ultrasonic sensors, and data acquisition instrument were used to complete the acoustic measurement experiment.

**Figure 14:**
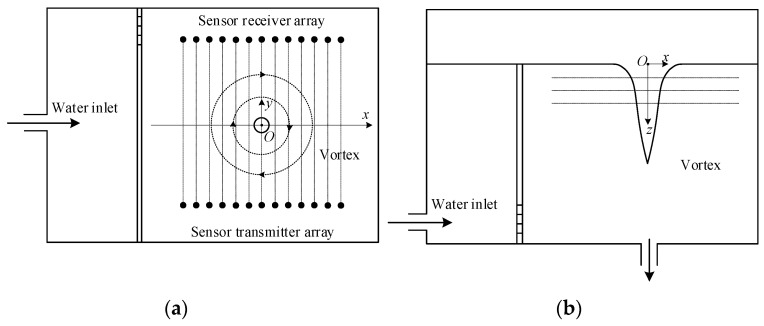
Experimental arrangement. (**a**) Top view; (**b**) Front view.

Figure 14 introduces the coordinate system and the experimental plane used in the study. The *x-O-y* plane is set on a horizontal plane, and the center of the circle corresponding to the bottom outlet is taken as the origin. The water depth is kept at 0.2 m, and the distance between the ultrasonic transmitter and receiver arrays is 0.24 m. Due to the fact that the ultrasonic sensor used in this experiment is a cylindrical device with a diameter of 0.035 m (as shown in Figure 13b), to prevent mutual interference among the elements and to align the transmitting and receiving arrays, the distance between the centers of each element is designed to be 0.045 m.

On the same horizontal plane, a total of 13 measurement points, ranging from −0.06 m to 0.6 m, are set every 0.01 m along the x-direction. There are three measurement planes at z = 0.03 m, z = 0.05 m, and z = 0.07 m below the horizontal plane in the z-direction, respectively (Figure 14).

The acoustic measurement experiment includes several steps. Firstly, a blank experiment was conducted in water to measure the propagation time of the acoustic signal in the absence of fluid flow. Subsequently, a fluid circulation device was activated, and the transmitter array emitted the set sound signal, which passed through the vortex and was then received by the receiver array. Due to the influence of the vortex on the sound signal, there was a positive or a negative time delay at the receiver end. The velocity distribution on the plane was thus obtained by inversion based on the collected time delay.

#### 4.2.2. Comparison and Analysis

**Figure 15:**
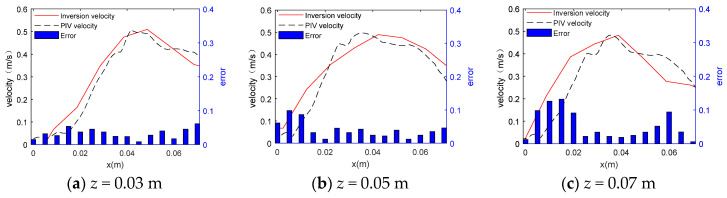
Biaxial column diagram of velocity distribution and absolute error on different measurement planes.

The velocity data measured by PIV and obtained by inversion were compared in the same coordinate system, and the absolute error between the two was calculated. The details are shown in Figure 15.

According to Figure 15, the change trend of the inversion velocity is similar to that of the PIV velocity. Both curves characterize the maximum tangential velocity and vortex core radius data.

The error is relatively large in the range of *x* = 0 to *x* = 0.01 m, and it increases with the increase of the *z* value. The reason is that there is an air column at the center of the vertical vortex. At the junction of air and water, the sound signal produces refraction and reflection effects, weakening the energy of the sound signal, affecting the signal-to-noise ratio and reducing the accuracy of acoustic measurement.

In the range of *x* = 0.03 to *x* = 0.05 m, where the maximum tangential velocity occurs, the absolute error between the inversion velocity and the PIV velocity is extremely small. The maximum tangential velocity and vortex core radius that this experiment focuses on occur within this range, so the influence of the air column on this experiment can be ignored.

It is worth mentioning that compared with that in the velocity curve measured by the acoustic method, the maximum tangential velocity range is more concentrated in the PIV velocity curve. Moreover, the decrease in its slope to both sides is also larger, indicating that the PIV measurement method has stronger dynamic resolution.

The error between the two methods was also calculated using PIV measurement data as the true value. The data are shown in Table 8 and Table 9.

According to Table 8, the absolute and relative errors in the maximum tangential velocity between the acoustic method and the PIV method are less than ±0.01 and within 10%, respectively. This indicates that the acoustic measurement method based on ultrasonic sensors is effective in measuring the velocity of the vertical vortex and highly accurate in measuring the maximum tangential velocity.

According to Table 9, the errors in the vortex core radius between the acoustic method and the PIV method increase, compared with those in Table 8. It is because the inversion of the flow velocity from the time delay is based on the assumption that the vortex is axisymmetric and the velocity is uniform. However, the real vortex is obviously irregular. As a result, errors in the vortex core radius increase.

The vortex velocity data obtained by two measurement methods were compared, and the error of the acoustic measurement data was calculated using PIV measurement data as standard values. The laboratory results verified the feasibility and accuracy of the acoustic measurement method for the velocity parameters of the vertical vortex.

## 5. Conclusions

The vertical vortex at the flood discharge outlet of hydraulic structures may cause serious harm and economic losses. Measuring the velocity of the vortex can help with the development of effective methods for vortex treatment. In this article, an acoustic measurement method for the vertical vortex was developed based on its sound propagation characteristics. Firstly, the PIV method was used to measure vortex data, and the acoustic measurement method was used to numerically simulate the data. The results verify the feasibility of the acoustic measurement method. Subsequently, CFD simulation was conducted at the flood discharge outlet of the Three Gorges Dam, and CFD data were used for numerical simulation of the acoustic measurement method. The flow velocity obtained by acoustic inversion was compared with that simulated by CFD. Finally, acoustic measurement experiments of the simplified vertical vortex model were conducted with the help of ultrasonic sensors. The experimental findings further verify the feasibility and accuracy of the acoustic measurement method.

The main conclusions of this article are drawn as follows:

1. The acoustic measurement method for the vertical vortex proposed in this article can effectively measure the velocity distribution and key parameters of the vortex, such as maximum tangential velocity and vortex core radius. PIV measurement data verify the effectiveness of the acoustic measurement method. The error of the maximum tangential velocity obtained is within 10%.
2. A reasonable arrangement of acoustic instruments based on the on-site environment at the flood discharge outlet of hydraulic structures can help create a real-time measurement system for the vertical vortex. The CFD numerical simulation and simplified model experiments of the vertical vortex both demonstrate that the acoustic measurement method can accurately measure the velocity parameters of the vertical vortex.
3. The accuracy of the acoustic measurement method is related to the velocity field reconstruction algorithm. Compared with existing measurement methods, the acoustic measurement method has better accuracy in measuring the maximum tangential velocity of the vertical vortex, but lower accuracy in measuring the size parameters.

In future research, the algorithm should be optimized to adapt to more forms of vortexes, thus improving the response speed of the acoustic measurement method to non-maximum flow velocity. Furthermore, field experiments should be conducted in hydraulic structures.

## Figures and Tables

**Table 1:** Vortex parameters derived from PIV measurements.

Plane	Maximum Tangential Velocity (m/s)	Vortex Core Radius (m)
z = 0.03 m	0.5074	0.0417
z = 0.05 m	0.4964	0.0360
z = 0.07 m	0.4827	0.0354

**Table 2:** Maximum tangential velocity data by PIV method and acoustic method.

Plane	Maximum Tangential Velocity (m/s)		Error	
	PIV Method	Acoustic Method	Absolute Error	Relative Error
z = 0.03 m	0.5074	0.4941	−0.0133	−2.69%
z = 0.05 m	0.4967	0.5007	0.0040	0.81%
z = 0.07 m	0.4827	0.4724	0.0103	2.18%

**Table 3:** Vortex core radius data by PIV method and acoustic method.

Plane	Vortex Core Radius (m)		Error	
	PIV Method	Acoustic Method	Absolute Error	Relative Error
z = 0.03 m	0.0417	0.0455	0.0038	9.11%
z = 0.05 m	0.0360	0.0368	0.0009	17.25%
z = 0.07 m	0.0354	0.0371	0.0056	11.45%

**Table 4:** Scales of the model.

Scale	Parameter
Flat scale	$\lambda_{l}=100$
Vertical scale	$\lambda_{h}=100$
Velocity scale	$\lambda_{v}=\lambda_{h}^{1/2}=10$
Water movement time scale	$\lambda_{t}=\lambda_{l}/\lambda_{v}=10$
Flow scale	$\lambda_{Q}=\lambda_{l}\lambda_{h}\lambda_{v}=100,000$

**Table 5:** CFD velocity data of the vertical vortex at the flood discharge outlet.

Plane	Maximum Tangential Velocity (m/s)	Vortex Core Radius (m)
z = 5 m	5.625	8.81
z = 10 m	5.131	7.56
z = 15 m	4.793	6.15

**Table 6:** Inversion velocity data of the vertical vortex at the flood discharge outlet.

Plane	Maximum Tangential Velocity (m/s)	Vortex Core Radius (m)
z = 5 m	5.576	9.90
z = 10 m	5.221	7.75
z = 15 m	4.919	6.45

**Table 7:** Errors in vortex data.

Plane	Maximum Tangential Velocity		Vortex Core Radius	
	Absolute Error	Relative Error	Absolute Error	Relative Error
z = 5 m	−0.049	−0.88%	1.14	12.50%
z = 10 m	−0.61	1.75%	1.06	18.12%
z = 15 m	0.126	2.62%	0.85	22.63%

**Table 8:** Maximum tangential velocity data by PIV method and acoustic experimental method.

Plane	Maximum Tangential Velocity (m/s)		Error	
	PIV Method	Acoustic Method	Absolute Error	Relative Error
z = 0.03 m	0.5074	0.5099	0.0025	0.49%
z = 0.05 m	0.4964	0.4889	−0.0075	−1.51%
z = 0.07 m	0.4827	0.4823	−0.0004	−0.08%

**Table 9:** Vortex core radius data by PIV method and acoustic experimental method.

Plane	Vortex Core Radius (m)		Error	
	PIV Method	Acoustic Method	Absolute Error	Relative Error
z = 0.03 m	0.0417	0.0486	0.0069	16.55%
z = 0.05 m	0.0360	0.0423	0.0063	17.50%
z = 0.07 m	0.0354	0.0388	0.0034	9.60%

## Data Availability

Data are contained within the article.

## References

[1] Yue Z., Chen H., Tian Z. (2023). Study on Hydraulic Characteristics of a Horizontal Eddy-elimination Barries Eliminating Vertical Vortex in Front of Sluice Gate. Water Resour. Power.

[2] Chen Y.L., Wu C., Ye M., Zhou Q. (2005). Research for flow pattern in intake of hydroelectric station. J. Hydro Dyn. (Ser. A).

[3] Lund F., Rojas C. (1989). Ultrasound as A Probe of Turbulence. Phys. D Nonlinear Phenom..

[4] Li H.-F., Chen H.-X., Ma Z., Zhou Y. (2008). Experimental and Numerical Investigation of Free Surface Vortex. J. Hydro Dyn. (Ser. B).

[5] Sun H.L., Liu Y.K., Zhang H.Y., Liu J.J. (2017). Experimental studies on characteristics of vortex formed in front of radial gate. Hydro Sci. Eng..

[6] Ru S., He X., Dai G. (1993). Experimental investigation on steady three-dimensional vortex flow field through horizontal orifices. J. Hydro Dyn. (Ser. B).

[7] Wang Y. (2011). Mechanical Characteristics and the Control Measurement for the Vertical Vortex.

[8] Rajendran V.P., Constantinescu S.G., Patel V.C. Experiments on flow in a model water-pump intake sump to validate a numerical model. Proceedings of the Fluids Engineering Division Summer Meeting, ASME.

[9] Rajendran V.P., Constantinescu S.G., Patel V.C. (1999). Experimental validation of a numerical model of flow in pump-intake bays. J. Hydraul. Eng..

[10] Rajendran V.P., Patel V.C. (2000). Measurement of vertices in model pump-intake bay by PIV. J. Hydraul. Eng..

[11] Guo M., Tang X., Li X., Wang F. (2019). Stereo PIV measurement of vortex in a model pump intake. IOP Conf. Ser. Earth Environ. Sci..

[12] Lv Y. (2021). Study on Evolution Characteristics and Control Measures of Vertical Vortex of Fupingbei in Guangyangba Reach of the Three Gorges Fluctuating Backwater Area.

[13] Yuta O., Harutaka H., Taku N. (2024). Spatial super-resolution based on simultaneous dual PIV measurement with different magnification. Exp. Fluids.

[14] Ruck S., Arbeiter F., Digel L. (2023). Laser Doppler and Hot-Wire Anemometry Measurements along V-shaped raised Ribs in a square Channel. Tech. Mess. Sensoren Gerate Syst..

[15] Manneville S., Maurel A., Roux P., Fink M. (1999). Characterization of a large vortex using acoustic time-reversal mirrors. Eur. Phys. J. B Condens. Matter Phys..

[16] Lillberg E., Alin N., Fureby C. (2000). Computational Study of Wakes behind Submarines and Surface Ships.

[17] Barber C., Lauchle G.C., Capone D.E. Water tunnel acoustic measurements using a time reversal mirror. Proceedings of the ASME International Mechanical Engineering Congress and Exposition, (IMECE 2001).

[18] Barber C., Lauchle G.C. (2004). Source level measurements of a cavitation noise source in a water tunnel using a time reversal mirror. J. Acoust. Soc. Am..

[19] de Rosny J., Tourin A., Derode A., Roux P., Fink M. (2005). Weak localization and time reversal of ultrasound in a rotational flow. Phys. Rev. Lett..

[20] Zhao W., Hu H., Fan S. (2018). Research on Flow-Acoustic Coupling of Transducer Disturbing Effect on Ultrasonic Flow meter. Meas. Control Technol..

[21] Constantinescu G.S., Patel V.C. (2000). Role of Turbulence Model in Prediction of Pump-Bay Vortex. J. Hydraul. Eng..

[22] Lai Y.G., Weber L.J., Patel V.C. (2003). A non-hydrostatic three-dimensional numerical model for hydraulic flow simulation-Part I: Formulation and Verification. J. Hydraul. Eng..

[23] Lai Y.G., Weber L.J., Patel V.C. (2003). A non-hydrostatic three-dimensional numerical model for hydraulic flow simulation-Part II: Validation and Application. J. Hydraul. Eng..

[24] Li S.H., Lai Y.G. (2004). Validation of a three-dimensional numerical model for water—Pump intakes. J. Hydraul. Res..

[25] Sakai T., Eguchi Y., Monji H., Ito K., Ohshima H. (2008). Ohshima H 2008 Heat Transfer. J. Hydraul. Eng..

[26] Suerich-Gulick F., Gaskin S.J., Villeneuve M., Parkinson É. (2014). Etienne Parkinson. Free surface intake vortex: Scale effects due to surface tension and viscosity. J. Hydraul. Res..

[27] Huang G.B., Shi M., Hu H. (2021). Numerical Simulation Study of the Vortex Phenomenon in front of the Surficial Discharge Sluice. Hydropower New Energy.

[28] Fuster D., Bagué A., Boeck T., Le Moyne L., Leboissetier A., Popinet S., Ray P., Scardovelli R., Zaleski S. (2009). Simulation of primary atomization with an octree adaptive mesh refinement and VOF method. Int. J. Multiph. Flow.

[29] Jiang X.L., Yang T., Zou Q.P., Gu H.B. (2018). Flow Separation and Vortex Dynamics in Waves Propagating over A Submerged Quarter-circular Breakwater. China Ocean. Eng..

[30] Shen C.Y., Yang R.G., Qing S., He S.H. (2023). Vortex analysis of water flow through gates by different vortex identification methods. J. Hydrodyn..

[31] Rankine W.J.M. (1858). Manual of Applied Mechanics.

[32] Manneville S., Maurel A., Bottausci F., Petitjeans P. (2000). Acoustic characterization of a stretched vortex in an infinite medium. Vortex Structure and Dynamics.

[33] Manneville S., Prada C., Tanter M., Fink M., Pinton J.-F. (2001). Ultrasound propagation through a rotational flow: Numerical methods compared to experiments. J. Comput. Acoust..

[34] Yu M., Fan D., Zhang Y., Liu H. (2023). Numerical simulation and experimental study of sound propagation in underwater steady vortex field. Tech. Acoust..

[35] Manneville S., Robres J.H., Maurel A., Petitjeans P., Fink M. (1999). Vortex dynamics investigation using an acoustic technique. Phys. Fluids.

[36] Gabel T., Mitra H., Williams D., Koeck F., Mónico R.O., Alba K. (2022). Incompressible flow through choke valve: An experimental and computational investigation. J. Fluids Struct..

[37] Zhao G., Lu J., Visser P.J., Vrijling K. (2011). Change of River Regime in River Systems: A Case Study of Shashi Reach in the Middle Yangtze River in Post-TGD (Three Gorges Dam) Period. Proceedings of the Fourth Yangtze Forum.

[38] Tang X.L., Wang F.J., Li Y.J., Cong G.H., Shi X.Y., Wu Y.L., Qi L.Y. (2011). Numerical investigations of vortex flows and vortex suppression schemes in a large pumping-station sump. Proc. Inst. Mech. Eng. Part C J. Mech. Eng. Sci..

[39] Verret D., LeBoeuf D. (2022). Dynamic characteristics assessment of the Denis-Perron dam (SM-3) based on ambient noise measurements. Earthq. Eng. Struct. Dyn..

[40] Duan W.G., Hou D.M., Wang C.H., Hu H., Tang X.F. (2019). Hydraulic prototype observation and analysis of Three Gorges Dam discharge structures. J. Water Resour..

[41] Yu S.D., Deng H., Chen X. (2004). Hydraulic safety monitoring of flood discharge structures in the Three Gorges Dam. Hydropower Autom. Dam Monit..

